# Phthalate Ester Contamination in Intensively Managed Greenhouse Facilities and the Assessment of Carcinogenic and Non-Carcinogenic Risk: A Regional Study

**DOI:** 10.3390/ijerph16162818

**Published:** 2019-08-07

**Authors:** Tingting Ma, Wei Zhou, Like Chen, Peter Christie, Yongming Luo, Peng Wu

**Affiliations:** 1 Institute of Hanjiang, Hubei University of Arts and Science, Xiangyang 441053, China; 2 Key Laboratory of Soil Environment and Pollution Remediation, Institute of Soil Science, Chinese Academy of Sciences, Nanjing 210008, China; 3 School of Civil Engineering and Architecture, Hubei University of Arts and Science, Xiangyang 441053, China; 4 Shanghai Research Institute of Chemical Industry, Shanghai 200062, China; 5 Jiangsu Rainfine Environmental Science and Technology Co. Ltd., Nanjing 210000, China

**Keywords:** central China, health risks, phthalate esters, plastic greenhouses

## Abstract

The contamination status and the potential carcinogenic and non-carcinogenic health risks from six phthalate esters (PAEs), nominated as priority pollutants by the United States Environmental Protection Agency (USEPA), were investigated in 40 typical greenhouses in three large-scale intensive greenhouse production areas in Jingmen city, Hubei province, central China. The total concentrations of PAEs in 40 soil samples and 80 vegetable samples ranged from 919 ± 134 to 7015 ± 475 µg kg^−1^ (dry weight, DW), and from 387 ± 63, to 11,683 ± 1313 µg kg^−1^ (DW), respectively. No carcinogenic risk was detected. The heat-map of the hazard quotient (HQ) values indicates the non-carcinogenic risks to children from di-n-butyl phthalate (DBP), at two sampling sites out of the 40, and from diethylhexyl phthalate (DEHP) (20 to young children and three to older children and adults) at 23 of the sites. The contamination risk from PAEs at Pengdun is of concern because only two of the 14 sampling sites selected there showed the non-carcinogenic risk to humans was unclear. The results of this study help to close a long-term knowledge gap resulting from a shortage of experimental data on PAE contamination in intensive greenhouse vegetable production in central China. The inclusion of DEHP in the Chinese list of priority pollutants is recommended, due to its increasing contamination and risk. This study provides valuable information for protected agricultural soil management and risk avoidance. It is a timely reminder to take PAE contamination and associated health risks into consideration, during the planning and introduction of intensively-managed greenhouse production systems.

## 1. Introduction

Greenhouse protected vegetable production avoids unsuitable conditions in the natural environment, to some extent by controlling growth conditions. It can extend the length of the annual growing season and increase yields, compared with traditional outdoor cultivation. The total area of protected vegetable production in China reached 39,000 km^2^ in 2016 and the yield of greenhouse vegetables was 252 million tonnes, accounting for 30.5% of the total yield of vegetables, and generating a net income of 570 billion yuan [1].

The use of plastic mulching film in China has increased greatly as greenhouse production systems have developed. Agricultural plastic film production currently accounts for 63% of total production of worldwide sales [2]. However, as a result of the unregulated uses of agricultural films, the release of phthalate esters (PAEs), during their use and degradation in protected crop production, has led to negative impacts on soil quality, as well as potential adverse effects on national food safety and human health [3].

High concentrations of PAEs are added to many polymers to increase their flexibility, in some cases up to 50% [4], and the concentrations in plastic mulching films are relatively high (40–60%) [5]. There is no strong physical or chemical bonding between the polymer and the plasticizers. The PAEs can readily be lost during the manufacture, use, and disposal of the plastics [6]. This results in pollution of soils, air, and waters [7,8,9,10,11]. PAEs can inhibit seedling germination and lead to plant antioxidant system disorders, damage to chloroplast ultrastructure, reduced fruit quality [12,13], and changes in soil microbial communities [14]. PAEs are a major public health concern because some are endocrine disrupters, carcinogens, teratogens, and mutagens, and their exposure potentially carries risks to of fertility damage, cancer, and the incidence of various chronic diseases, such as coronary disease and diabetes [15,16,17,18,19]. The United States Environmental Protection Agency (USEPA) has determined that the phthalic acid esters dimethyl phthalate (DMP), diethyl phthalate (DEP), di-n-butyl phthalate (DBP), butylbenzyl phthalate (BBP), diethylhexyl phthalate (DEHP) and dioctyl phthalate (DOP) are priority pollutants [20]. Health risk assessments have been considered necessary, given the recognition of PAE toxicity [21]. There has been increasing number of concerns regarding the exposure pathways of different PAEs, and the risks that PAEs may present to human health and the environment.

The Chinese government strongly supports the development of facility agriculture [22]. Central China supplies vegetables to millions of people so the effects of PAE contamination can have a greater impact on consumer. In 2012 the total area of greenhouse vegetable production in Hubei province was 1980 km^2^ and the harvested yield reached 4.6 million tonnes [23]. However, catastrophic flooding in July 2016 resulted in direct economic losses of 20 billion yuan to Hubei province and underlined the urgent need to accelerate the development of facility agriculture. Jingmen is a typical representative city of facility agricultural production in Hubei province and a major vegetable source for Wuhan, the provincial capital city. Contamination of lakes and groundwater by PAEs has long been a matter of concern [24,25] but there have been no detailed studies on PAE contamination of protected vegetable soils in Hubei province.

We investigated PAE contamination in three large-scale vegetable production facilities in Hubei province, central China, at Zilingpu, Zhanghe, and Pengdun in Jingmen city. Forty soil samples and 80 vegetable samples (40 foliar samples and 40 vegetable fruit samples) were collected from 40 typical production greenhouses. The contamination status of the six target PAE compounds and their risk assessments, both carcinogenic and non-carcinogenic, were evaluated based on the analysis of these samples.

## 2. Materials and Methods

### 2.1. Study Area

Hubei province is located in central China between 29°01′53′′–33°6′47′′ N and 108°21′42′′–116°07′50′′ E and adjacent to Anhui, Chongqing, Shaanxi, Jiangxi, Hunan and Henan provinces. The total area of Hubei province is 185,900 km^2^, accounting for 1.94% of the total land area of China. The landform is an incomplete basin surrounded by mountains to the east, west, and north. The province has 13 prefecture-level administrative regions, including 12 prefecture-level cities and one autonomous prefecture, with a permanent population of 59.17 million. The total cultivated land area is about 33,487 km^2^, but the arable land per capita is only 533 m^2^, lower than the national average. Facility agriculture is an important channel for agricultural development in the province. By 2017, the area of facility vegetables had reached 1600 km^2^, more than 30% of the total area of vegetable cultivation.

Jingmen is a typical city in Hubei province with protected crop production facilities. It lies near the middle and lower reaches of the Hanjiang river and has a total area of 12,400 km^2^, at an altitude of 96 m above mean sea level. The local climate is northern subtropical monsoon, with four distinct seasons. The annual average temperature is 16.6 °C and the annual rainfall is about 1000 mm, with 1690 h of sunshine annually. By 2015 the total area of greenhouse vegetable was 400 km^2^ and is expected to continue to increase [26]. The production facilities comprise plastic greenhouses within which seedlings are covered by 2–4 additional layers of plastic film, in order to maintain optimum temperature and moisture conditions, thereby resulting in large amounts of mulching film usage. Cost-conscious farmers often select cheap agricultural film of low quality. There is no regulation of the use of plastic film or its recycling, so that polymers remaining in the soil can lead to contamination with PAE compounds.

Three relatively large-scale vegetable production facilities were selected. The first is Luoyuan vegetable professional cooperative in Bajiao village, Zilingpu town, Dongbao district, which operates as a family farm. Its main business comprises vegetable production, sales, and related services. The second facility, Shuangfu Ecological Agriculture Development Co. Ltd., is situated in Tandian village, Zhanghe new district, and is adjacent to Zhanghe reservoir. The company has an area of 20,000 m^2^ and provides services such as grain purchase, storage, processing and marketing. The main business is vegetable cultivation and processing together with other organic agriculture activities and by 2014 the total vegetable planting area was 0.2 km^2^. The third facility is in Pengdun village, Shipai town, Zhongxiang city. Pengdun village has 0.27 km^2^ of vegetable production greenhouses that are engaged mainly in off-season vegetables and specialty vegetables. Over 10,000 tonnes of various vegetables are harvested each year. General production information on the three study areas is summarized in Table S1.

### 2.2. Standards and Reagents

The standard solutions (2 mg mL^−1^) of the six target PAE pollutants, recommended in the list of priority pollutants by USEPA, comprising DMP, DEP, DBP, BBP, DEHP, and DOP, mixed in hexane and benzyl benzoate (BB) dry reagent (100 mg), used as an internal standard, were all obtained from AccuStandard Corporation (New Haven, CT, USA). A certified reference material (CRM) 136-100 (BNAs-Clay 1) was purchased from RT Corporation (Laramie, WY, USA). The organic solvents acetone and hexane (both high performance liquid chromatography grade) were purchased from Tedia Company (Fairfield, OH, USA). The packing materials Na_2_SO_4_ (reagent grade), neutral Al_2_O_3_ (400 mesh, reagent grade), neutral silica gel (100 to 200 mesh), and H_2_SO_4_ (guaranteed reagent) for glassware washing were obtained from Tianjin Yongda Chemical Reagent Co. Ltd., Tianjin, China. The packing materials were dried separately in a muffle furnace at 400 °C for 6 h and stored in desiccators before use [27].

### 2.3. Sampling

**Figure 1:**
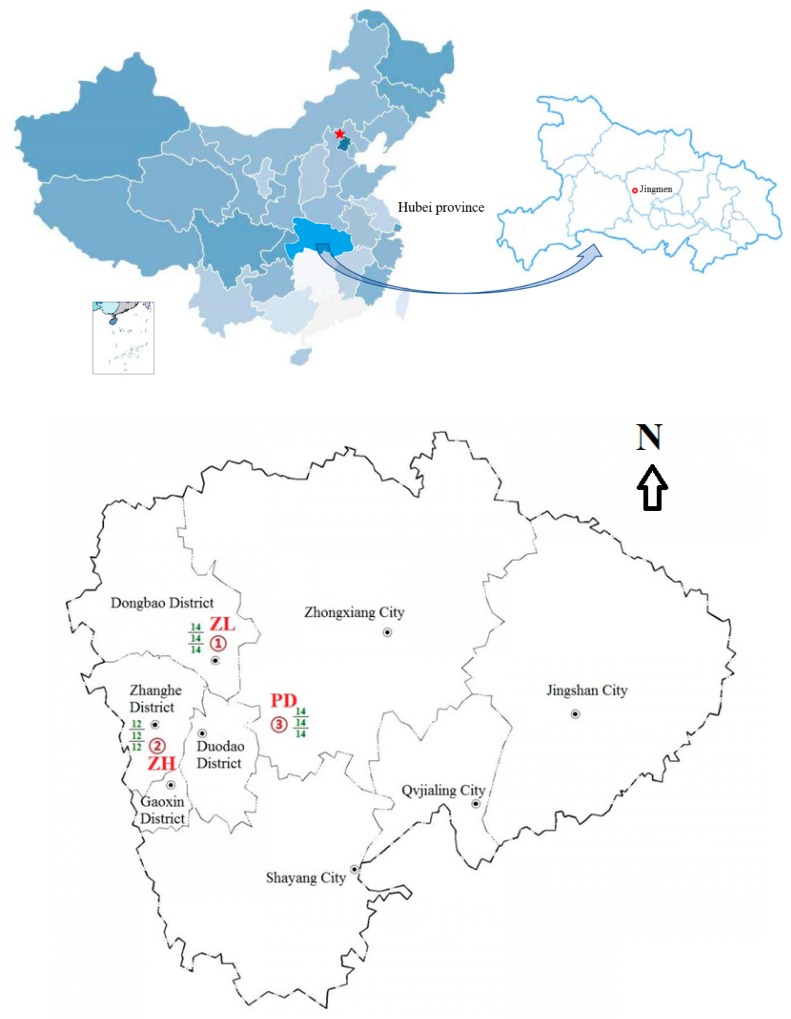
Geographical locations of sampling site details in an administrative map (1:6,000,000) of Jingmen city. ZL (14/14/14), Zilingpu (14 fruit sample/14 leaf sample/14 soil sample); ZH (12/12/12), Zhanghe (12 fruit sample/12 leaf sample/12 soil sample); PD (14/14/14), Pengdun (14 fruit sample/14 leaf sample/14 soil sample).

There are 190 vegetable production greenhouses across the three study areas. In 77 greenhouses at Zilingpu, 22 grew yellow cucumber, 18 grew green tomato, 16 grew green chili, and 21 red chestnut pumpkin. In 39 greenhouses at Zhanghe, 11 grew long purple eggplant, 13 Hangzhou chili, and 15 cauliflower. In 74 greenhouses at Pengdun, 24 grew tomato, 22 mini-cucumber, 12 eggplant and 16 screw chilies. A total of 120 samples, comprising soils and vegetables, were collected from 40 non-adjacent vegetable production greenhouses out of 190 at the three study areas in November 2018 (Figure 1, Table S1), including 4 yellow cucumber, 3 green tomato, 3 green chili, and 4 red chestnut pumpkin at Zilingpu; 4 long purple eggplant, 4 Hangzhou chili, and 4 cauliflower at Zhanghe; 4 tomato, 4 mini-cucumber, 3 eggplant, and 3 screw chili at Pengdun. Each of the 120 samples was a composite of five individual samples collected in a quincunx pattern from the 40 greenhouses screened, giving a total of 600 individual samples collected. Soil samples were collected from the surface (0–15 cm depth) using a soil corer. The foliar and vegetable fruit samples were selected and cut randomly from non-adjacent and non-marginal plants, and analyzed separately in triplicate. Vegetable samples were washed with tap water, rinsed with distilled water, and wiped dry with paper tissue before freeze drying in a Free Zone 2.5-Liter Freeze Dry System (Labconco Corporation, Kansas City, MO, USA). Soil samples were freeze-dried directly to maintain a constant weight before sampling determination, following their transport to the laboratory in linen sampling bags. The pH value, electrical conductivity (EC), organic matter content (SOM), and mechanical composition of the soils, from three different areas, were determined following the methods of Lu [28] and listed in Table S2.

### 2.4. Sample Analysis and Quality Control

Glassware was washed by strictly following the procedure provided by Ma et al. [27] prior to analysis. Dried soil was ground and sieved (60 mesh), and each replicate of the dried plant samples was homogenized in liquid nitrogen prior to storage at −20 °C for subsequent analysis.

Ten g of soil (dry weight, DW) or 2 g (DW) of frozen dried vegetable sample were extracted with a total volume of 70 mL acetone: Hexane (1:1 *v*/*v*) three times in a water bath at 25 °C and reduced in the flask by rotary evaporation to 1–2 mL (350 mbar, 40 °C water bath, 80 rpm) after centrifugation at 1500 rpm. Column chromatography purification was conducted in a glass column (1 × 26 cm) with Na_2_SO_4_, neutral Al_2_O_3_ and neutral silica gel (from bottom to top) with acetone:hexane (1:4, *v*/*v*) before collection and reduction to <1 mL by rotary evaporation as described above [27].

The analysis was conducted with a Model 7890BGC-5977A MSD gas chromatograph-mass spectrometer (Agilent Technologies, Santa Clara, CA, USA). The MS operating parameters were as follows: Ionization under electron impact (EI) mode at 230 °C with detector voltage at 1.012 kV; scan mode under selected ion monitoring mode and transfer line 280 °C. A DB-5 (30 m × 0.25 mm × 0.25 µm) fused silica capillary column with helium (purity > 99.999%) as a carrier gas at 1.2 mL min^–1^ was used to separate the compounds. The injector temperature was set at 250 °C. The GC oven temperature was programmed as follows: Initial temperature of 50 °C held for 1 min, increased at a rate of 15 °C min^–1^ to 200 °C, held for 1 min, then increased at a rate of 8 °C min^–1^ to 280 °C, then held for 3 min. Post-run was at 290 °C for 1 min. Under-selected ion monitoring mode, non-pulse injection, with a volume of 1 μL in split-less mode, was carried out [27]. Three whole procedure blanks and one CRM 136-100 were included with every 24 samples for quality assurance/quality control (QA/QC) during analysis to ensure analytical reliability [27]. Limits of detection (LOD) of the six PAEs ranged from 0.10 to 0.35 µg L^–1^ and limit of quantification (LOQ) of the spiked test soil ranged from 50 to 120 µg kg^–1^.

### 2.5. Health Risk Assessment

The carcinogenic and non-carcinogenic risks of the six target PAEs were evaluated according to the risk assessment guidelines recommended by USEPA [29]. Daily intakes of vegetables, and the different paths of absorption from soils, have also been considered in the evaluation of potential health risks to consumers [30]. Here, farmers and their children are exposed to soil PAE contamination risk by dermal and inhalation contact, as well as through soil ingestion and consumed vegetables harvested from local greenhouses. Dietary intake data, recommended by the Environment Agency and Department of Environment, Food and Rural Affairs, Danish Nationwide Dietary Survey, in Contaminated Land Exposure Assessment (CLEA), London with slight modification and divided into age groups 0–6 (refer to the data of 1–4 of CLEA) and 7–70 in the vegetable consumption estimates [30]. The hazard quotient (*HQ*) value was used to quantify the non-carcinogenic risk assessment induced by the six target PAEs. The *HQ* values were obtained by summing the average daily dosage (*ADD*, mg kg^−1^ d^−1^) from different exposure pathways, namely intake, ingestion, dermal absorption and inhalation and dividing by the corresponding reference dose (*RfD*, mg kg^−1^ d^−1^) of each PAE. The hazard index (*HI*) is equal to the sum of the *HQ* values of the individual PAE compounds. An *HI* < 1 indicates no significant risk of non-carcinogenic effects and an *HI* > 1 indicates that non-carcinogenic effects may occur [31]. Vegetable intake, soil ingestion, dermal absorption, and inhalation were calculated as follows [32],

$$ADD_{\mathrm{int}ake}=\frac{C_{fruit}\times IRF\times EF\times ED\times CF}{BW\times AT}$$

$$ADD_{ingestion}=\frac{C_{soil}\times IRS\times EF\times ED\times CF}{BW\times AT}$$

$$ADD_{dermal}=\frac{C_{soil}\times SA\times AF\times ABS\times EF\times ED\times CF}{BW\times AT}$$

$$ADD_{inhale}=\frac{C_{soil}\times EF\times ED\times 1000}{PEF\times AT}$$

$$HI=\sum_{i=6}HQ=\sum_{i=6}\frac{ADD_{\mathrm{int}ake}+ADD_{ingestion}+ADD_{dermal}+ADD_{inhale}}{RfD}$$

where, *C_fruit_*, PAE concentration in edible vegetable parts (mg kg^−1^ DW); *IRF*, daily vegetable intake; *EF*, exposure frequency (days y^−1^); *ED*, exposure duration (production duration of different plastic greenhouses); BW, body weight; *AT*, average time, (365 × *ED* for *HQ* calculation, lifetime (25,550 days) for carcinogenic risk assessment); *CF*, conversion factor (10^−6^ kg mg^−1^); *C_soil_*, PAE concentration in soil samples (mg kg^−1^ DW); *IRS*, soil ingestion rate; *SA*, soil surface area; *AF*, soil adherence factor; *ABS*, fraction of contaminant absorbed dermally from the soil; *PEF*, particle emission factor.

Cancer risk is acceptable when *CR* falls within the range of threshold values (10^−6^ to 10^−4^). In the carcinogenic risk assessment, *CR* (unitless, carcinogenic risk) was calculated by the following equation:

$$CR_{j}=ADD_{j}\times SF$$

where, *SF* (kg^−d^ mg^−1^) is the slope factor of the carcinogen of exposure route, derived from dose-response studies and comprising oral slope factors (SFO) for ingestion, dermal contact ((mg kg^−1^ d^−1^)^−1^), and inhalation unit risk ((mg m^−3^)^−1^). Detailed parameters for health risk assessment are listed in Table 1.

### 2.6. Statistical Analysis

PAE values are presented as the mean values of five replicates ± the standard deviations of five replicate samples in the same sampling greenhouses. All data were processed using Microsoft Excel 2013. The resulting differences were assessed by one-way analysis of variance (ANOVA), followed by the least significance difference (LSD) test at *p* < 0.05 to compare the soil, foliar, or vegetable fruit samples between different study areas. The large number of samples collected in this study, with high variability, will undoubtedly provide a wealth of information for analysis and investigation. But more importantly, in most cases there may be correlations between these variables, which increases the complexity and inconvenience of the analyses. If each parameter is analyzed separately, the isolated analysis, rather than comprehensive, tends to blindly reduce some indicators, which leads to the loss of much information and/or wrong conclusions. Therefore, a principal component analysis (PCA) was adopted to reduce the loss of information contained in the original indices, while reducing the data volume so as to achieve the purpose of comprehensive analysis of the data collected. Cluster analysis is a clustering and classification process based on the similarity between the arrays. This makes it easier to explore and discover data structures in large amounts of data. A hierarchical clustering method is adopted here to facilitate the partitioning of datasets at different levels so as to form a tree-like clustering structure and to interpret the results in a visual way. PCA of PAEs, in edible parts of plant samples in the three study areas, presented in 3D and 2D, cluster analysis of PAEs in foliar samples and a heatmap of HQ values were plotted. The correlation analysis was conducted using Pearson correlation at *p* < 0.05 and *p* < 0.01 with two-tailed test of significance. Pearson correlation analysis was conducted to compare different target PAEs in different selected greenhouses.

## 3. Results and Discussion

### 3.1. Soil Contamination with the Six Target PAEs

**Figure 2:**
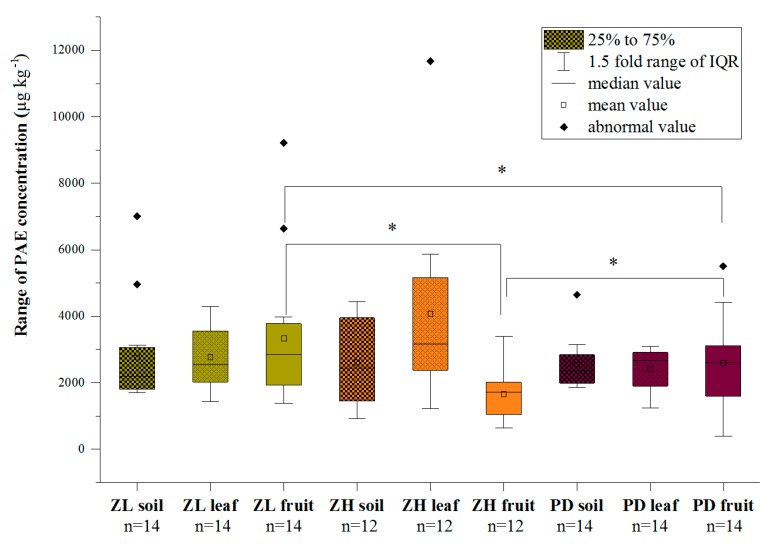
Concentration range of PAEs in 120 samples from 40 selected plastic greenhouses in three study areas. *: significant difference at *p* < 0.05 compared with the same sampling group. Refer the denotes to Figure 1.

The total concentrations of the target PAEs in the surface soils, at the three study areas (Tables S3–S5 and Figure 2), ranged from 1699 ± 314 to 7015 ± 475 µg kg^−1^ DW at Zilingpu, 919 ± 135 to 4435 ± 522 µg kg^−1^ DW at Zhanghe, and 1867 ± 281 to 4655 ± 1091 µg kg^−1^ DW at Pengdun. Omitting the outlier values in the different areas, the concentrations and ranges of the six target PAEs followed the sequence: Zhanghe (*n* = 12) > Zilingpu (*n* = 14) > Pengdun (*n* = 14) but were not significantly different (*p* > 0.05) (Figure 2). DBP and DEHP were the major soil congeners in all the plastic greenhouses sampled at Zilingpu, with only traces of BBP detected. Soil concentrations of DMP, DEP, and DOP were all relatively low, (<50 µg kg^−1^ DW). Even in greenhouses, where the same vegetables were grown, the contamination levels of different PAE compounds, especially DBP and DEHP, varied widely (Table S3). The average values of soil total PAE levels of four vegetable species followed the sequence: Cucumber (yellow) > pumpkin (red chestnut) > tomato (green) > chili (green). Similar trends in the six target pollutants were observed at Zhanghe and Pengdun (Tables S4 and S5). No soil BBP was detected at Zhanghe, and the average soil total PAE levels of three vegetable species followed the order: eggplant (purple long) > chili (Hangzhou) > cauliflower (Table S4). Soil BBP was also undetected at Pengdun, where the average soil total PAE levels followed the sequence: Tomato > cucumber (mini) > eggplant > chili (screw) (Table S5).

The soil PAE composition patterns (Figure S1a) show that, DBP and DEHP accounted for >95% of the total PAE values in samples from Zilingpu. The composition of DBP ranged from 69% to 91% and that of DEHP, from 9% to 29%. At Zhanghe the percentage sum of DBP and DEHP was also >95% (Figure S1b), ranging from 14% to 86% (DBP) and 12% to 84% (DEHP). Similar values were found in PD soils, with DBP ranging from, 55% to 82% and DEHP from 16% to 42% (Figure S1c).

Due to the high cropping index, large agricultural inputs, and the closed environment in the greenhouses, can lead to high health risks because of the accumulation of various pollutants. This is not consistent with sustainable development of production [38]. Environmental degradation in China over the past three decades has encouraged numerous studies on the distribution of soil PAEs and their risk assessment, and this has led to a fairly comprehensive assessment of soil PAE contamination nationwide [39]. The levels of PAEs in Chinese soils are generally at the high end of the global range and some are higher than the Grade II limits of the Environmental Quality Standard for soils in China, i.e., 10,000 µg total PAEs kg^−1^ soil DW [39]. Without detailed information in central China, higher total soil concentrations of PAEs (sum of DMP, DEP, DBP and DEHP) have been found in greenhouses in north China in recent years, for example, in northeast China (2110 ± 320 µg kg^−1^ DW) and Shouguang (a major vegetable production area) in Shandong (7350–33,390 µg kg^−1^ DW) [40,41]. Intensive greenhouse vegetable production is highly developed in north and northeast China because of the relatively cold weather and short growing seasons in the field. However, in central China (Hubei, Hunan, Henan, and Jiangxi provinces), production conditions are more favorable and there has been little concern over the environmental effects of greenhouse production, and intensive greenhouse production using plastic greenhouses has developed rapidly.

### 3.2. Plant Contamination Levels of the Six Target PAEs

Plant total concentrations of the target PAEs at the three study areas were determined separately in the leaves and vegetable fruits (Tables S3–S5 and Figure 2). Total foliar PAE concentrations ranged on average from 1432 ± 763 to 4290 ± 607 µg kg^−1^ DW, following the sequence: Cucumber (yellow) > tomato (green) > pumpkin (red chestnut) > chili (green) at Zilingpu (Table S3), 1222 ± 276 to 11,683 ± 1313 µg kg^−1^ DW following the order: Cauliflower > eggplant (purple long) > chili (Hangzhou) at Zhanghe (Table S4) and 1238 ± 56 to 3091 ± 805 µg kg^−1^ DW following the order: eggplant > chili (screw) > cucumber (mini) > tomato at Pengdun (Table S5). However, the edible parts of vegetables are the most important, due to the potential for pollutant transfer through the food chain. In vegetable fruit samples, based on the average values of the same vegetable species, the total PAE concentrations determined ranged from 1377 ± 99 to 9216 ± 632 µg kg^−1^ DW following the sequence: pumpkin (red chestnut) > tomato (green) > cucumber (yellow) > chili (green) at Zilingpu (Table S3), 642 ± 46 to 3392 ± 396 µg kg^−1^ DW following the order: Cauliflower > eggplant (purple long) > chili (Hangzhou) at Zhanghe (Table S4), i.e., the same trend as in the leaves, and 387 ± 63 to 5506 ± 3330 µg kg^−1^ DW following the sequence: Tomato > chili (screw) > eggplant > cucumber (mini) at Pengdun (Table S5). Omitting the outlier values in different areas from Figure 2, the mean concentration and range of the six target PAEs in leaves and fruit were, respectively, in the sequence: Zhanghe (*n* = 12) > Zilingpu (n = 14) > Pengdun (*n* = 14) with no significant difference (*p* > 0.05), and Pengdun (*n* = 14) > Zilingpu (n = 14) > Zhanghe (*n* = 12) with a significant difference (*p* < 0.05).

DBP and DEHP were the major congeners present in both foliar and vegetable fruit samples from Zilingpu, with foliar and fruit DMP and foliar BBP all < 1 µg kg^−1^ DW, fruit BBP below the detection limit, and the levels of both DEP and DOP ≤ 30 µg kg^−1^ DW (Table S3). BBP was undetected in both leaves, and vegetable fruits at Zhanghe and the other congeners (except DBP and DEHP) were < 50 µg kg^−1^ DW (Table S4). At Pengdun, foliar BBP was not detected and DMP in vegetable fruits and DMP, DEP and DOP in the leaves were < 1 µg kg^−1^ DW, except for DBP and DEHP, which were much higher (Table S5). Within the same category of vegetable only chili could be compared across the three study areas and followed the sequence Pengdun (screw chili) > Zilingpu (green chili) > Zhanghe (Hangzhou chili). No significant differences were observed between cucumber at Zilingpu (yellow cucumber) and Pengdun (mini-cucumber), tomato at Zilingpu (green tomato) and Pengdun (tomato), as well as eggplant at Zhanghe (purple long eggplant) and Pengdun (eggplant).

The DBP and DEHP contamination levels also differed greatly in greenhouses in which the same vegetable species were grown. The PAE composition pattern results (Figure S1a) show that DBP and DEHP accounted for >95% of the total PAE values in both leaf and vegetable fruit samples from Zilingpu. The composition range of DBP was 39% to 98% and 47% to 88%, and that of DEHP was from <3% to 59% and 12% to 50% in leaf, and vegetable fruit samples, respectively. Although the percentage of DOP at Zilingpu was higher than other samples in other greenhouses in other areas, it was still ≤3% in 75% of foliar samples determined and all vegetable fruit samples. The percentage sum of DBP and DEHP was >95% except in two chili (Hangzhou) samples (Figure S1b), ranging from 16 to 75% and 11 to 37% (DBP) and from 21 to 82% and 62 to 89% (DEHP) in foliar, and vegetable fruit samples, respectively. However, at Pengdun, the percentages of DOP in foliar samples of all vegetables sampled were >3%. Except in cucumber (mini) 2, the sum of DBP and DEHP was also >80% of the total PAE concentration (Figure S1c). The percentage ranges of DBP in foliar and vegetable fruit samples were between 14% and 81% and <3% to 94%; and those of DEHP were between 14% and 40% and 6% and 98%, respectively.

Total concentrations of PAEs in vegetables of 3000 ± 2100 to 6940, 7160 or 8090 µg kg^−1^ DW have been reported from other studies [30,42,43,44]. In contrast to soil results, DEHP, DBP, and DOP have been found to be the most abundant congeners, accounting for >90% of total PAEs, consistent with the situation in Pengdun, where the percentages of DOP in vegetables were significantly (*p* < 0.05) higher than in soils. However, no leafy vegetables have been planted in our three study areas and these might show higher contamination status in greenhouse production.This should be a focus in future investigations. Thus, the quality of vegetables, grown in soils with PAE contamination, may deteriorate, and under the stress of the PAEs, the seedlings might become vulnerable in species, such as winter melon, cucumber, and chili [45,46]. The control of PAE contamination, and subsequent remediation by different methods, requires investigation.

**Figure 3:**
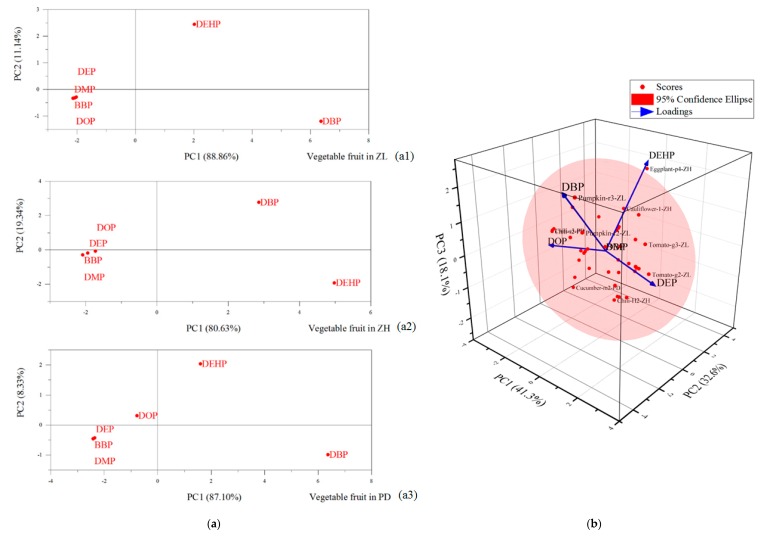
Principal component analysis (PCA) of PAEs in edible parts of samples collected from three study areas. (**a**) 2D PCA results of the six target pollutants in three individual study areas; (**a1**) 2D PCA results of the six target pollutants at Zilingpu; (**a2**) 2D PCA results of the six target pollutants at Zhanghe; (**a3**) 2D PCA results of the six target pollutants at Pengdun; and (**b**) 3D PCA results of all vegetables determined. Pumpkin-r2-ZL, the second sampling greenhouse planted pumpkin (red chestnut)/red chestnut pumpkin in Zilingpu; Pumpkin-r3-ZL, the third sampling greenhouse planted pumpkin (red chestnut)/red chestnut pumpkin in Zilingpu; Tomato-g2-ZL, the second sampling greenhouse planted tomato (green)/green tomato in Zilingpu; Tomato-g3-ZL, the third sampling greenhouse planted tomato (green)/green tomato in Zilingpu; Eggplant-p4-ZH, the forth sampling greenhouse planted eggplant (purple long)/purple long eggplant in Zhanghe; Chili-H2-ZH, the second sampling greenhouse planted chili (Hangzhou)/Hangzhou chili in Zhanghe; Cauliflower-1-ZH, the first sampling greenhouse planted cauliflower in Zhanghe; Chili-s2-PD, the second sampling greenhouse planted chili (screw)/screw chili in Pengdun; Chili-s3-PD, the third sampling greenhouse planted chili (screw)/screw chili in Pengdun; Cucumber-m2-PD, the second sampling greenhouse planted cucumber (mini)/mini-cucumber in Pengdun.

From the results in Figure 3a, Tables S6 and S7, the PCA results of vegetable fruit samples from Zilingpu the contribution rate of the first principal component was 88.86%, and the characteristics of factor variables values in yellow cucumber 4, green tomato 3, green chili 1, green tomato 1, green tomato 2, yellow cucumber 2, and yellow cucumber 1 showed a high positive load, indicating that these samples can represent the characteristics of the target pollutants in the area quite well. In the first principal component analysis, DBP showed the largest difference, followed by DEHP, while DEP, DMP, BBP, and DOP showed small differences. The contribution rate of the second principal component was 11.14%, and the characteristics of factor variable values in red chestnut pumpkin 1, red chestnut pumpkin 4, green chili 2, yellow cucumber 3, red chestnut pumpkin 3, red chestnut pumpkin 2, and green chili 3 had high positive loads. In the second principal component analysis, DEHP showed the largest difference, followed by DBP, while DEP, DMP, BBP, and DOP showed small differences.

As shown in the results in Figure 3a, Tables S6 and S7, in the PCA results of vegetable fruit samples from Zhanghe saw a contribution rate of the first principal component was 80.63% and the characteristics of factor variable values in purple long eggplant 3, purple long eggplant 1, purple long eggplant 2, cauliflower 3, cauliflower 1, and purple long eggplant 4 showed high positive loads. Similar to Zilingpu, in the first principal component analysis, DBP also showed the largest difference, followed by DEHP, while DEP, DMP, BBP, and DOP showed small differences. The contribution rate of the second principal component was 19.34% and the characteristics of factor variables values in cauliflower 4, cauliflower 2, Hangzhou chili 4, Hangzhou chili 1, Hangzhou chili 2, and Hangzhou chili 3 showed a high positive load. In the second principal component analysis, DBP showed the largest difference, followed by DEHP, while DEP, DMP, BBP, and DOP showed small differences.

From the results in Figure 3a, Tables S6 and S7, in the PCA results of vegetable fruit samples from Pengdun, the contribution rate of the first principal component was 87.10%, and the characteristics of factor variable values in screw chili 2, screw chili 3, screw chili 1, tomato 3, eggplant 2, eggplant 1 and mini-cucumber 1 showed high positive load. In the first principal component analysis, DBP showed the largest difference, followed by DEHP, and then by DEP, BBP, and DMP, while DOP showed the smallest difference. The contribution rate of the second principal component was 8.33%, and the characteristics of factor variables values in mini-cucumber 3, eggplant 3, tomato 4, tomato 2, tomato 1, mini-cucumber 2, and mini-cucumber 4 showed high positive loads. In the second principal component analysis DEHP showed the largest difference, followed by DBP, DEP, BBP, and DMP, while DOP showed the smallest difference.

From the results in Figure 3b, Table S6 and S8, in the PCA results of all the vegetable fruit samples the contribution rate of the first principal component was 41.31%, and the characteristics of factor variables values in Tomato-g2-ZL, Tomato-g3-ZL, Tomato-g1-ZL, Chili-g1-ZL, Cucumber-y2-ZL, Chili-g3-ZL, Chili-g2-ZL, Cucumber-y4-ZL and Pumpkin-r3-ZL showed high positive load. The contribution rate of the second principal component was 32.59% and the characteristics of factor variable values in Eggplant-p4-ZH, Cauliflower-1-ZH, Cauliflower-2-ZH, Cucumber-m4-PD, Cauliflower-4-ZH, Cucumber-y3-ZL, Cauliflower-3-ZH, Eggplant-p1-ZH and Eggplant-p2-ZH showed high positive load. The contribution rate of the third principal component was 18.10%, and the characteristics of factor variable values in Pumpkin-r3-ZL, Eggplant-p4-ZH, Tomato-4-PD, Pumpkin-r2-ZL, Cucumber-y3-ZL, Cauliflower-1-ZH, Tomato-g3-ZL, Cucumber-y4-ZL, and Cucumber-m4-PD showed high positive load.

According to Figure 3b, in the three principal component analysis DEHP showed the largest difference, followed by DBP and DEP, while DOP, BBP, and DMP showed the smallest difference. In the first principal component coordinate, DEP showed the largest difference, followed by DOP, DBP, and DEHP, while BBP and DMP showed the smallest difference. In the second principal component coordinate DEHP showed the largest difference, followed by DBP and DOP, and BBP, DEP, and DMP, which showed the smallest difference. In the third principal component coordinate DBP showed the largest difference, followed by DEHP, and DOP, BBP, DEP and DMP showed the smallest difference.

PCA results of six target PAEs, from vegetable fruit samples in individual study areas, indicate that DBP and DEHP are recognized as the most representative pollutants in all vegetable fruit samples (Figure 3a). One principle component (PC1) has an eigenvalue >1 explained >80% of the total variance in each sampling area (Table S6). In the first principal component analysis of each study area (Figure 3a), DBP showed the largest difference, followed by DEHP without exception. However, the likely explanation for their difference in vegetable fruits largely relies on the source of the target PAEs, forming the basis for follow-up investigations from this study. A major dietary source of PAEs reported are vegetables which contribute to 45.5% of total exposure, including beans, eggplant, luffa, tomatoes, cucumber, melon, Chinese cabbage, radish, celery, leek, and cauliflower [47]. PAEs can remain inside the human body at certain levels with constant intake from the edible fruits of these vegetables, which indicates that these vegetables can transfer the contaminant PAEs through the human food chain. However, the order of magnitude of the transfer remains unknown.

**Figure 4:**
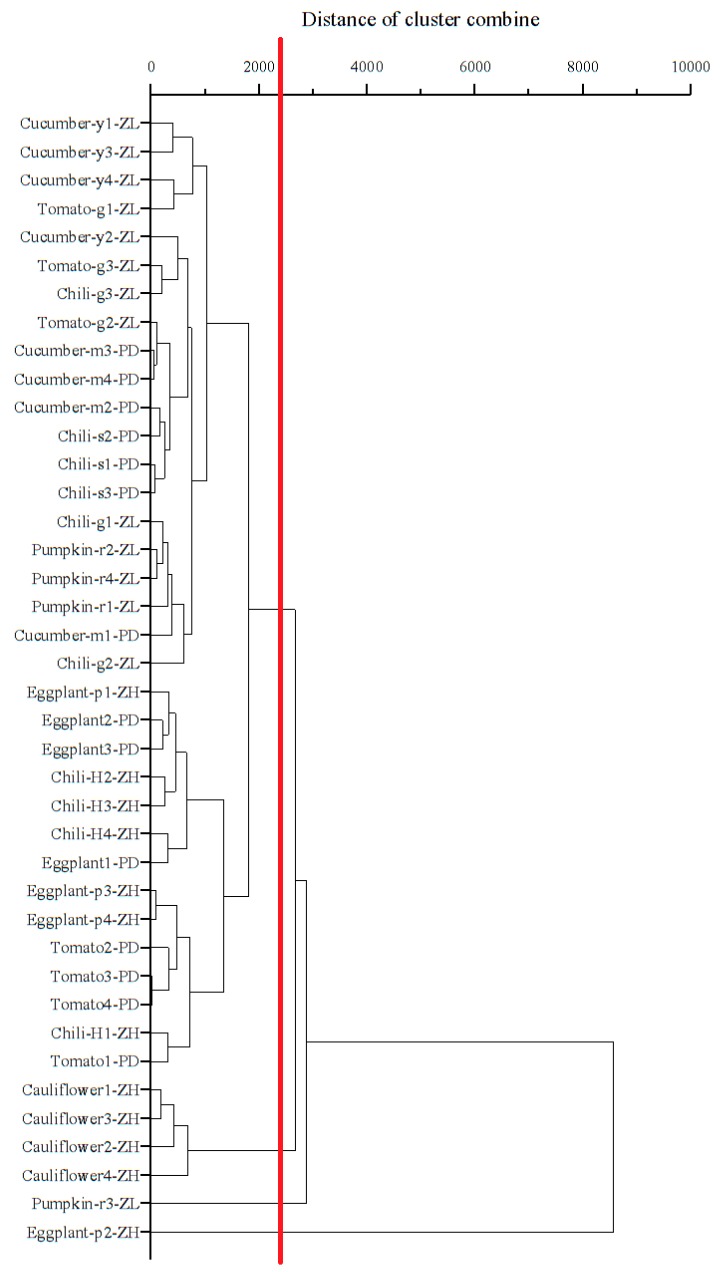
Cluster analysis of PAEs in vegetable foliar samples collected from the three study areas. Cucumber-y1-ZL, the first sampling greenhouse planted cucumber (yellow)/yellow cucumber in Zilingpu; Tomato-g1-ZL, the first sampling greenhouse planted tomato (green)/green tomato in Zilingpu; Chili-g1-ZL, the first sampling greenhouse planted chili (green)/green chili in Zilingpu; Pumpkin-r1-ZL, the first sampling greenhouse planted pumpkin (red chestnut)/red chestnut pumpkin in Zilingpu; Eggplant-p1-ZH, the first sampling greenhouse planted eggplant (purple long)/purple long eggplant in Zhanghe; Chili-H1-ZH, the first sampling greenhouse planted chili (Hangzhou)/Hangzhou chili in Zhanghe; Cauliflower-1-ZH, the first sampling greenhouse planted cauliflower in Zhanghe; Tomato-1-PD, the first sampling greenhouse planted tomato in Pengdun; Cucumber-m1-PD, the first sampling greenhouse planted cucumber (mini)/mini-cucumber in Pengdun; Eggplant-1-PD, the first sampling greenhouse planted eggplant in Pengdun; Chili-s1-PD, the first sampling greenhouse planted chili (screw)/screw chili in Pengdun.

In this study, cluster analysis was used to comprehensively evaluate the similarity and distance of PAE contamination, in foliar samples at each sampling site, to reflect the spatial distribution characteristics of the total target PAEs. Cluster analysis of the foliar sample data (Figure 4) shows that the three most important clusters (see the red line) comprised eggplant (purple long) 2 at Zhanghe, pumpkin (red chestnut) 3 at Zilingpu, and all 4 cauliflower leaves at Zhanghe. The contamination status of the samples classified in the same cluster is similar. The farther the distance between clusters, the greater the contamination difference. Thus, the contamination status of eggplant (purple long) 2 at Zhanghe, pumpkin (red chestnut) 3 at Zilingpu, and all 4 cauliflower leaves at Zhanghe, are more similar and much higher than all the other samples. Compared with the concentration values, the total concentrations in these foliar samples of the three main clusters were all >4200 µg PAEs kg^−1^ DW.

### 3.3. Non-Carcinogenic and Carcinogenic Risks from the Six Target PAEs

**Figure 5:**
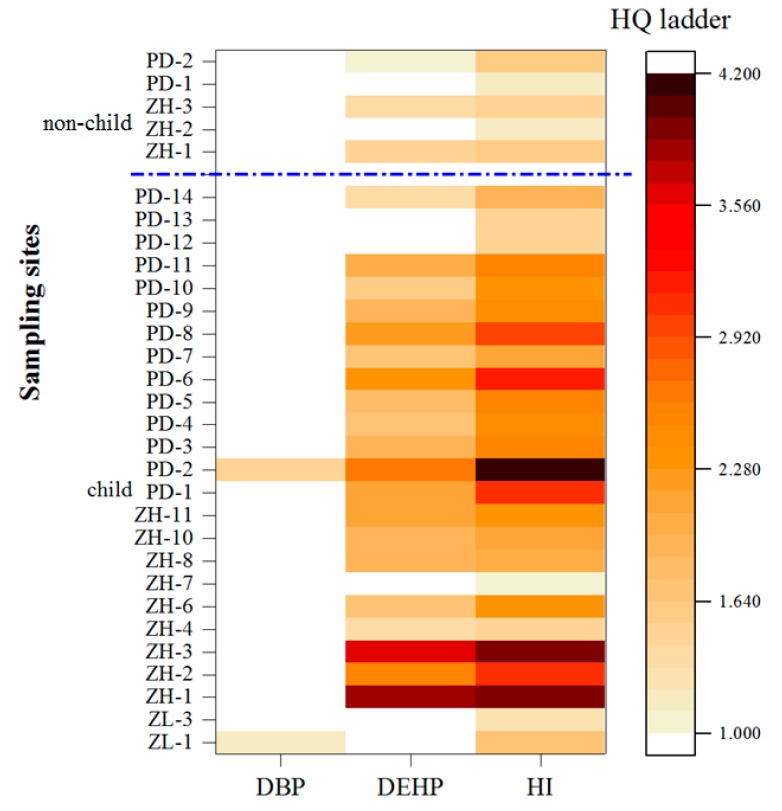
Heatmap of HQ values of all samples collected from the three study areas. Letters are abbreviations of study area; and numbers after the dash are the sequences of sampling sites.

The calculated non-carcinogenic health risks are shown in Figure 5. Only DBP and DEHP were present in adequate concentrations to represent the non-carcinogenic risk, but not in the whole population. The occurrence of non-carcinogenic risk to older children and adults was predicted in 25 of the 40 greenhouses sampled, in three greenhouses at Zhanghe and two at Pengdun. All sampling sites at Pengdun represented some non-carcinogenic risk to children aged <6 years of age. Zhanghe had 75% of its sampling sites in a similar situation, and Zilingpu had one greenhouse of seven showing a risk to children <6 years. Ingestion and dermal absorption are the most important sources of PAEs in this risk assessment rather than intake from edible parts of vegetables or soil inhalation. Similar conclusions were drawn by Wang et al. [35] who investigated the distribution of, and health risks from, PAEs in street dusts of Xi’an city, northwest China. They found that ingestion and dermal absorption of dust particles were the main pathways of exposure. The non-carcinogenic risk to humans in the current study were caused by DEHP, rather than DBP, while the risk to children was higher than the risks of the substance to older children and adults, and these findings also agree with Wang et al. [35]. However, Chen et al. [44] concluded that dietary exposure to PAEs, through vegetable intake, was higher than the total exposure from other foodstuffs, inhalation, or dermal absorption. All pathways of PAE transfer to the human body and causing health risks along the food chain and the control of PAEs in soils and greenhouse vegetables require further investigation.

**Figure 6:**
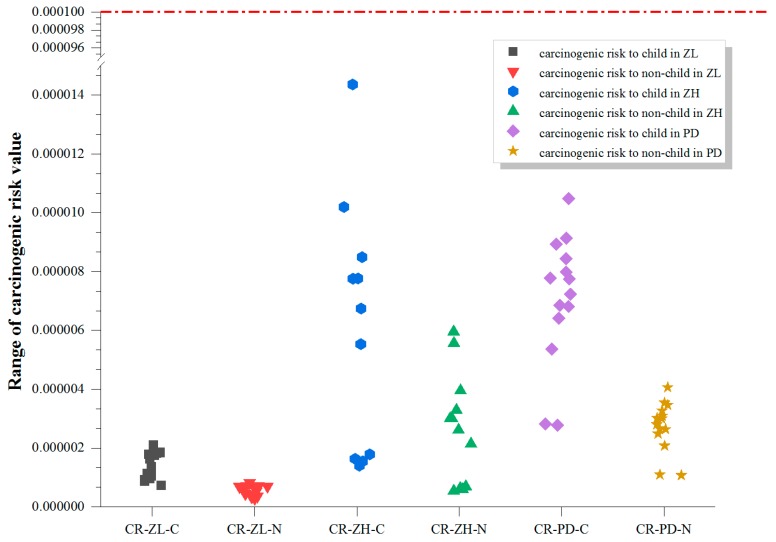
Carcinogenic risk in sampling sites of two of the three study areas. CR-ZL-C denotes carcinogenic risk to young children at Zilingpu, and CR-ZL-N denotes carcinogenic risk to older children and adults at Zilingpu. Other details, see Figure 1.

Our calculated carcinogenic risks were only due to DEHP (Figure 6), but all the values were below the threshold value of 10^−4^, suggesting that the cancer risks, from human exposure to DEHP in the greenhouses sampled, were all acceptable. The risk may have been overestimated because the process of vegetable washing, cooking with oil, and digestion can alter the contamination on the surface/inside of the food, and food intake comprises only a very small percentage (<one thousandth) in the non-carcinogenic and carcinogenic health risk values. However, the prediction in this study is still useful. In a study of contamination and human health risks from PAEs in agricultural soils on the Sanjiang Plain, northeast China, the non-carcinogenic risk was negligible but the carcinogenic risk from DEHP exceeded the threshold limit [32]. Management strategies are urgently required to control pollution by PAEs, especially from DEHP, particularly because of the contaminants’ capacity to accumulate and increase the carcinogenic risks. DEHP is frequently detected, contamination levels increase together with its health risks growing throughout China. The addition of DEHP to the priority pollutant list (currently DMP, DBP and DOP) is recommended.

### 3.4. Source Identification of Target PAEs

The sums of soil, vegetable leaf and vegetable fruit PAEs, in each production area of plastic greenhouses, have been combined to conduct the Pearson correlation analysis (Table S9). At Zilingpu the correlations between DMP and DEHP (0.05), DMP and DOP (0.01), and DEHP and DOP (0.05), were significant. At Zhanghe, the correlations between DMP and DEP (0.01), DMP and DOP (0.01), and DEP and DOP (0.01), were significant. At Pengdun, only the correlation between DMP and DEP (0.01) was significant. Significant correlations indicate similar environmental sources or environmental behavior between pollutants. DMP and DEP, in addition to their small molecular weights, which enable them to degrade readily, are also used as solvents in insecticide sprays. DEHP and DOP are also used as solvents, and are PAE congeners with high molecular weights and are not readily degraded.

The most frequently detected PAE congeners in most investigated soils, dusts, sediments, and plants are DBP and DEHP [39,48], and this corresponds with the results of the current study (Figure S1a–c). It has been reported that the occurrence of the two most abundant congeners is associated with the use of plastic film (especially plastic mulching film in agricultural soils) in urban, rural, and agricultural soils [39]. Soil PAE concentrations are impacted by the color and residual volume of the plastic [49]. In addition to the use of plastic films, wastewater irrigation, and application of fertilizers and pesticides, are also major sources of PAEs in soils [44]. However, the concentrations of the six target PAEs in different films, irrigation waters, fertilizers and pesticides need to be determined form the main part of our future study on source analysis.

The general order of production areas in terms of total PAE contamination was Zhanghe (*n* = 12) > Zilingpu (*n* = 14) > Pengdun (*n* = 14) in the soils, Zhanghe (*n* = 12) > Zilingpu (*n* = 14) > Pengdun (*n* = 14) in the leaves and Pengdun (*n* = 14) > Zilingpu (*n* = 14) > Zhanghe (*n* = 12) in the vegetable fruits. Significant differences were found only between vegetable fruit samples in the three study areas (*p* < 0.05), but there appeared to be differences in contamination between Zhanghe and Pengdun. Vegetable production has been practiced for 5 years, and 8 years, respectively at Zhanghe and Pengdun, and the total cultivation areas, the use of greenhouse films and their thickness, and the types of mulching films used, vary greatly. The total years of intensive operation can result in a direct time-dependent increase in total PAE accumulation [50], thus contamination is likely to be higher at Pengdun.

At Zhanghe and Pengdun, polyethylene (PE) and polyolefin (PO) membranes are used as greenhouse plastic films. Both PE and PO films are sources of microplastics in soils and it has been estimated that 63,000–430,000 and 44,000–300,000 tonnes of microplastics may be added annually to agricultural land in Europe, and North America, respectively [51,52]. PE membranes are the simplest and the most widely used polymer materials globally, and are regarded as suitable materials for contact with foods. Their characteristics of light texture and transparency, moisture-proof-ness, oxygen-, acid-, alkali-resistance, and excellent heat-sealing performance have broadened their range of application. PE is sensitive to environmental stress (chemical and mechanical effects) and has poor heat resistance with aging. In this respect, PO offers advantages. PO films are heat-shrinkable, newly-developed, and widely used environmentally friendly materials in Europe and North America. They are also in full compliance with the Food and Drug Administration of the United States (USFDA) and the United States Department of Agriculture (USDA) standards as excellent hot packaging materials. PO membranes are waterproof and are resistant to aging. This helps to minimize the incidence of diseases and insect pests, increasing the quality of vegetables, and reducing the costs of pesticide and herbicide applications. The use of PO membranes may explain the lower use of pesticides at Pengdun.

### 3.5. Management Strategies to Control PAE Contamination in the Study Areas

Based on our source identification information, several important factors that may contribute to pollution by target PAEs, are the type of mulching membrane and type of greenhouse plastic film (PE or PO), the amount of pesticide sprayed, the duration of greenhouse cultivation, the source of irrigation water, and the fertilizer application rates. Additional management strategies are required to control, or at least mitigate, the PAE pollution.

## 4. Conclusions

The total PAE concentrations in the soil, foliar, and vegetable fruit samples, from 40 sampled greenhouses, indicate that the contamination levels are significant. No leafy vegetables were grown at the three study areas but high levels of PAEs (>4200 µg PAEs kg^−1^ DW in eggplant (purple long) at Zhanghe, pumpkin (red chestnut) at Zilingpu and cauliflower leaves at Zhanghe) indicate that PAE contamination should be monitored along with the contamination of vegetable fruit samples with potentially toxic elements. Non-carcinogenic health risks to children <6 years old should be taken into account, especially at Zhanghe and Pengdun, while adults also appear to be at some risk. All the sampling sites at Pengdun, and 75% at Zhanghe, showed an HI > 1 to children <6 years old. The PCA results indicated that DBP and DEHP are the most representative pollutants in all the vegetable fruit samples collected. The control of PAEs in soils and greenhouse vegetables needs to be given high priority in intensively managed facilities in central China. The resulting data may provide a reference for the further development of greenhouse crop production, with appropriate pollution countermeasures for risk control and environmental management, and thus help to ensure the safety of food products in the future.

## Figures and Tables

**Table 1:** Parameters for calculation of health risk assessment.

Parameter		Young Children	Older Children and Adults		Unit	References
Age		0 to 6	7 to 70			Ma et al. [30]
Body weight (BW)		13.6	Female 57.3	Male 66.2	kg	Chinese MoH [33]
			61.75			
IRF (daily vegetable intake)	Carrot (not leafy)	0.125	0.054		g DW (kg BW day^−1^)	Ma et al. [30]
EF (exposure frequency)		Production frequency of plastic greenhouses (calculated from Table S1)			days year^−1^	Ma et al. [30]
		1/3 of adults	273 ZL, 303 ZH, 334 PD			
ED (Exposure duration)		Production duration of plastic greenhouses (see Table S1)			year	Ma et al. [30]
CF (conversion factor)		10^−6^			kg mg^−1^	USEPA [34]
AT (average time)		365×ED for hazard quotient (HQ), lifetime (25,550) for carcinogenic risk (CR)			days	Wang et al. [35]
IRS (soil ingestion rate)		200	100		mg day^−1^	USEPA [31]
SA (soil surface area)			5700		cm^2^ day^−1^	Wang et al. [35]
AF (soil adherence factor)			0.07		mg cm^−2^	Wang et al. [35]
ABS (fraction of contaminant absorbed dermally from the soil)			0.1		unitless	Wang et al. [35]
PEF (particles emission factor)			1.36 × 10^9^		m^3^ kg^−1^	Wang et al. [35]
SFO (oral slope factor of the carcinogen)	BBP	1.90 × 10^−3^			(mg kg^−1^ day^−1^)^−1^	Niu et al. [36]
	DEHP	1.40 × 10^−2^				
RfD (New Mexico Environment Department, NMED)	DMP	10			10^3^ µg (kg day)^−1^	NMED [37]
	DEP	0.8				
	BBP	0.2				
	DBP	0.1				
	DEHP	0.02				
	DOP	0.04				

Abbreviations: Dimethyl phthalate (DMP), diethyl phthalate (DEP), di-n-butyl phthalate (DBP), butylbenzyl phthalate (BBP), diethylhexyl phthalate (DEHP), dioctyl phthalate (DOP).

## Supplementary Materials

The following are available online at https://www.mdpi.com/1660-4601/16/16/2818/s1, Figure S1: The composition patterns of PAEs in samples collected from (a) Zilingpu (*n* = 52 = 14 fruit sample + 14 leaf sample + 14 soil sample). Cucumber-y1, the first sampling greenhouse planted cucumber (yellow)/yellow cucumber; Tomato-g1, the first sampling greenhouse planted tomato (green)/green tomato; Chili-g1, the first sampling greenhouse planted chili (green)/green chili; Pumpkin-r1, the first sampling greenhouse planted pumpkin (red chestnut)/red chestnut pumpkin. (b) Zhanghe (*n* = 36 = 12 fruit sample + 12 leaf sample + 12 soil sample). Eggplant-p1, the first sampling greenhouse planted eggplant (purple long)/purple long eggplant; Chili-H1, the first sampling greenhouse planted chili (Hangzhou)/Hangzhou chili; Cauliflower-1, the first sampling greenhouse planted cauliflower. (c) Pengdun (*n* = 52 = 14 fruit sample + 14 leaf sample + 14 soil sample). Tomato-1, the first sampling greenhouse planted tomato; Cucumber-m1, the first sampling greenhouse planted cucumber (mini)/mini-cucumber; Eggplant-1, the first sampling greenhouse planted eggplant; Chili-s1, the first sampling greenhouse planted chili (screw)/screw chili. Histogram heights <3% are hidden, Table S1: Overview usage information on plastic membranes and production mode at the three study areas, Table S2: Physicochemical properties of soils at the three study areas, Table S3: PAE concentrations in samples collected from Zilingpu (µg kg^−1^ dry weight, DW), Table S4: PAE concentrations in samples collected from Zhanghe (µg kg^−1^ DW), Table S5: PAE concentrations in samples collected from Pengdun (µg kg^−1^ DW), Table S6: Eigenvalues and contribution rates of PCA, Table S7: Eigenvectors of principal component analysis in Figure 3a, Table S8: Eigenvectors of principal component analysis in Figure 3b, Table S9: Pearson correlation analysis of PAEs in different samples.
