# Supplementary material for: Phthalate Ester Contamination in Intensively Managed Greenhouse Facilities and the Assessment of Carcinogenic and Non-Carcinogenic Risk: A Regional Study
Source: Int J Environ Res Public Health. 2019 Aug 7;16(16):2818. doi: 10.3390/ijerph16162818 (PMC6719942; doi:10.3390/ijerph16162818)
Supplement: Supplementary file 1 [file ijerph-16-02818-s001.pdf]

Table S1. Overview usage information on plastic membranes and production mode at the three study areas.

Site	Location	Planting area (m²)	Greenhouse film						
			Type	Thickness (mm)	Continuous Covering Time (a)	Breadth (m)	Consumption (t)	Recycle	Recycle Mode
ZL	Ziling Town	66,667	Composite	8–15	4	12–15	16–24	Yes	Unified recycle and reuse
ZH	Zhanghe Town	6400	PE (polyethylene)	>12	5	8	50	Yes	Unified recycle and reuse
PD	Pengdun Village	66,667	PO (polyolefin)	6–12	8	8	35	Yes	Unified recycle and reuse

Abb	Mulching Film						Productive Time
	Type	Thickness (mm)	Recycle	Residues	Recycle Time	Recycle Mode	
ZL	Black and double-sided mulch	14	Yes	No visible debris	After harvest	Manual and discard	Sep to May
ZH	Colored /weeding mulch	8–12	Yes	No visible debris	After harvest	Manual and discard	Aug to Nov & Feb to Jul
PD	Colored /weeding mulch	8–12	Yes	No visible debris	After harvest	Manual and discard	Aug to Dec & Jan to Jun

Table S2. Physicochemical properties of soils at the three study areas.

Location	Soil Type	pH	EC (μS cm^{-1})	Clay (%)	Silt (%)	Sand (%)	Organic matter (%)
ZL	Alfisols	5.27 ± 0.44	963 ± 96	12	13	75	1.42 ± 0.17
PD	Alfisols	6.32 ± 0.49	487 ± 62	17	10	73	1.67 ± 0.21
ZH	Alfisols	5.88 ± 0.35	351 ± 77	22	12	66	1.88 ± 0.11

Table S3. concentrations in samples collected from Zilingpu (μg kg^{-1} dry weight, DW).

Sampling Site	Soil							Leaf							Fruit						
	DMP	DEP	BBP	DBP	DEHP	DOP	PAEs	DMP	DEP	BBP	DBP	DEHP	DOP	PAEs	DMP	DEP	BBP	DBP	DEHP	DOP	PAEs
**1**	13±4	26±3	ND	6374±407	597±59	5±1	7015±475a	-	3±0	-	2337±431	1213±27	2±0	3556±458ab	-	18±4	ND	1037±24	852±35	10±0	1918±63f
**2**	15±5	22±3	ND	2587±244	496±169	4±1	3125±423c	-	3±0	-	1362±52	1377±18	1±0	2744±70c	-	22±3	ND	1081±68	263±25	11±2	1377±99g
**3**	11±3	24±2	ND	4405±457	507±184	7±0	4954±647b	-	3±0	-	2564±195	1565±212	-	4132±407a	-	17±2	ND	1591±111	1697±53	10±1	3316±168cd
**4**	15±2	24±6	ND	1632±31	459±21	6±1	2136±62d	-	2±0	-	1988±197	1666±60	-	3656±258a	-	21±3	ND	2101±78	1186±54	12±3	3321±139cd
**5**	18±5	28±3	ND	1353±41	321±84	2±0	1722±134e	-	5±1	-	1562±52	1767±36	-	3335±89b	-	26±8	ND	2434±431	713±63	16±3	3189±505cd
**6**	21±2	29±8	ND	2101±169	299±148	3±1	2454±329d	-	7±2	-	1929±39	1154±34	-	3090±75b	-	29±5	ND	1262±52	902±43	19±4	2212±104e
**7**	23±4	28±4	ND	2182±99	260±115	2±0	2495±223d	-	5±1	-	1263±363	989±23	1±0	2258±388cd	-	28±3	ND	2796±195	1132±14	18±3	3973±215c
**8**	11±2	35±3	-	1835±132	272±36	7±1	2160±175d	-	6±1	-	1773±148	576±59	1±0	2357±209cd	-	22±5	ND	1190±197	775±27	8±0	1996±229e
**9**	9±1	39±12	ND	1922±189	249±22	7±2	2227±227d	-	3±0	-	1030±594	399±169	-	1432±763d	-	21±6	ND	861±52	891±18	6±0	1780±76f
**10**	10±2	34±2	ND	1442±260	206±48	6±1	1699±314de	-	3±0	-	1229±609	776±184	1±0	2009±794d	-	22±5	ND	621±39	939±212	8±0	1590±257f
**11**	7±3	29±8	ND	1498±200	385±214	2±0	1921±426de	-	9±2	-	1377±407	387±21	2±0	1776±431d	-	13±2	ND	3190±363	570±60	10±2	3783±428cd
**12**	8±3	29±3	ND	2500±176	517±75	1±0	3055±258c	-	11±1	-	1577±244	354±84	1±0	1943±330d	-	13±2	ND	6500±148	111±36	11±1	6635±188b
**13**	7±2	27±8	ND	1255±65	499±41	1±0	1789±116e	-	10±1	-	3756±457	523±148	1±0	4290±607a	-	15±3	ND	8977±594	213±34	10±1	9216±632a
**14**	9±2	28±3	ND	1227±32	523±76	2±0	1790±114e	-	9±2	-	1677±31	429±115	-	2115±149cd	-	14±3	ND	2148±609	331±23	12±2	2505±637cde

Values are mean values of three replicates ± standard deviation; SD < 0.5 recorded as 0. ND: not detected; –, < 1. Sample numbers 1 to 4, yellow cucumber; 5 to 7, green tomato; 8 to 10, green chili; 11 to 14, red chestnut pumpkin. Different superscript letters indicate significant differences between different samples according to one-way analysis of variance followed by Tukey's test ($p < 0.05$).

Table S4. PAE concentrations in samples collected from Zhanghe (μg kg^{-1} DW).

Sampling Site	Soil							Leaf							Fruit						
	DMP	DEP	BBP	DBP	DEHP	DOP	PAEs	DMP	DEP	BBP	DBP	DEHP	DOP	PAEs	DMP	DEP	BBP	DBP	DEHP	DOP	PAEs
**1**	29±3	15±2	ND	519±72	3124±418	16±3	3704±498a	5±1	6±1	ND	547±128	2523±4446	8±2	3089±577e	-	13±2	ND	846±109	1087±377	30±5	1975±492bc
**2**	29±3	15±2	ND	2304±249	2072±264	15±3	4435±522a	5±1	6±1	ND	1580±260	10081±10487	10±2	11683±1313a	-	13±2	ND	476±101	891±39	33±4	1413±146de
**3**	28±3	15±2	ND	1364±106	2919±331	16±3	4343±445a	5±1	6±1	ND	400±131	800±141	11±3	1222±276g	-	15±2	ND	468±119	681±20	30±4	1194±145e
**4**	28±3	14±2	ND	416±40	1123±94	16±3	1597±141d	5±1	7±1	ND	495±9	834±85	10±3	1351±98g	-	13±3	ND	575±42	2774±347	30±4	3392±396a
**5**	11±3	20±2	ND	677±80	314±76	20±3	1042±165e	8±1	3±1	ND	651±46	1614±701	14±2	2289±751fg	-	20±2	ND	541±39	287±227	42±3	890±271f
**6**	11±3	21±1	ND	2782±0	1370±297	19±3	4205±500a	9±1	3±1	ND	380±52	2191±306	13±2	2594±360f	-	18±2	ND	390±6	191±35	43±3	642±46f
**7**	13±3	22±2	ND	2000±97	332±75	19±2	2833±180b	9±1	3±1	ND	475±42	1940±390	15±2	2442±436f	-	18±2	ND	1237±38	346±101	41±3	1643±144d
**8**	12±3	21±2	ND	449±65	1576±111	18±2	2077±184c	8±1	2±1	ND	347±62	2855±226	13±2	3225±291e	-	19±2	ND	476±57	281±8	43±3	819±69f
**9**	50±3	12±3	ND	483±74	362±53	12±3	919±135ef	3±1	4±1	ND	1373±26	3719±112	6±2	5106±142c	-	8±1	ND	469±20	1840±84	34±3	2350±108b
**10**	47±2	13±4	ND	532±94	1578±256	12±4	2182±360c	3±1	4±1	ND	1282±28	3390±183	7±2	4686±214d	-	8±2	ND	285±10	1500±190	36±3	1830±205c
**11**	47±2	13±2	ND	884±86	1725±221	13±3	2684±313bc	3±1	4±1	ND	1300±33	3902±138	6±2	5216±174c	-	9±1	ND	646±192	1355±66	35±3	2044±262c
**12**	49±2	13±3	ND	929±110	284±89	13±3	1289±207de	3±1	5±1	ND	1524±27	4332±129	8±2	5873±159b	-	8±2	ND	284±25	1469±78	36±3	1797±108c

Values are mean values of three replicates ± standard deviation; SD < 0.5 recorded as 0. ND: not detected; –, < 1. Sample numbers 1 to 4, purple long eggplant; 5 to 8, Hangzhou chili; 9 to 12, cauliflower. Different superscript letters indicate significant differences between different samples according to one-way analysis of variance followed by Tukey's test ($p < 0.05$).

Table S5. concentrations in samples collected from Pengdun (μg kg^{-1} DW).

Sampling Site	Soil							Leaf							Fruit						
	DMP	DEP	BBP	DBP	DEHP	DOP	PAEs	DMP	DEP	BBP	DBP	DEHP	DOP	PAEs	DMP	DEP	BBP	DBP	DEHP	DOP	PAEs
**1**	20±3	26±5	ND	2111±615	972±128	18±6	3147±757ab	-	-	ND	908±565	1415±50	-	2323±615abc	-	14±3	ND	712±51	626±54	203±36	1556±144h
**2**	18±4	22±6	ND	3392±952	1208±123	15±6	4655±1091a	-	-	ND	376±145	1329±44	-	1704±189c	-	13±6	ND	3554±257	615±20	229±33	4412±316bc
**3**	19±3	25±4	ND	1407±354	893±62	15±6	2358±429cde	-	-	ND	94±37	1151±59	-	1245±95d	-	13±5	ND	1829±1327	737±103	232±30	2811±1464abjh
**4**	19±2	25±5	ND	1718±94	789±14	17±5	2570±119c	-	-	ND	110±11	1128±46	-	1238±56d	-	11±5	ND	4260±3249	1020±49	214±26	5506±3330abf
**5**	13±2	19±4	ND	1627±378	833±101	13±5	2505±490bcd	-	-	ND	1784±10	111±20	-	1895±30c	-	6±4	ND	560±330	254±328	179±20	999±681h
**6**	13±3	19±5	ND	1985±120	1052±111	13±7	3084±246b	-	-	ND	2070±45	733±438	-	2803±483a	-	5±3	ND	131±21	83±26	168±14	387±63i
**7**	11±2	18±6	ND	1038±20	785±250	15±4	1867±281de	-	-	ND	1834±130	1209±90	-	3043±220a	-	5±3	ND	896±76	535±39	157±17	1594±13hj
**8**	12±3	18±2	ND	1775±136	1029±391	13±6	2847±537bc	-	-	ND	1809±13	1141±10	-	2950±23a	-	6±3	ND	303±122	1624±7	159±20	2092±152gj
**9**	16±4	14±3	ND	1170±53	896±32	16±6	2111±98e	-	-	ND	67±18	3024±787	-	3091±805ab	-	9±2	ND	1859±18	686±36	133±26	2688±251adfj
**10**	16±2	13±4	ND	1588±246	739±563	16±3	2372±818bce	-	-	ND	116±20	2497±492	-	2613±512ab	-	8±3	ND	1695±6	636±29	136±34	2476±73bf
**11**	15±3	12±3	ND	1266±238	920±400	17±3	2230±646bce	-	-	ND	334±373	2435±45	-	2769±418ab	-	8±3	ND	1815±46	569±7	130±17	2522±73bf
**12**	5±2	5±3	ND	1644±336	319±107	20±4	1993±152e	-	-	ND	1769±21	877±28	-	2647±50ab	-	3±2	ND	2045±86	855±38	303±23	3206±150ae
**13**	4±2	5±3	ND	1609±120	325±126	22±2	1964±253e	-	-	ND	2025±45	892±60	-	2917±105ab	-	4±2	ND	1921±147	851±66	334±29	3111±245ae
**14**	5±1	4±3	ND	1318±40	618±157	22±3	1967±205e	-	-	ND	1842±77	841±41	-	2683±118ab	-	3±2	ND	1913±80	846±1	316±27	3078±111ae

Values are mean values of three replicates ± standard deviation; SD < 0.5 recorded as 0. ND: not detected; –, < 1. Sample numbers 1 to 4, tomato; 5 to 8, mini-cucumber; 9 to 11, eggplant; and 12 to 14, screw chili. Different superscript letters indicate significant differences between different samples according to one-way analysis of variance followed by Tukey's test ($p < 0.05$).

Table S6. Eigenvalues and contribution rates of PCA.

Sampling site	Component	Eigenvalue	Contribution Rate	Cumulative Contribution Rate
Zilingpu	Principal component 1	12.441	88.86%	88.86%
	Principal component 2	1.5592	11.14%	100.00%
Zhanghe	Principal component 1	9.6756	80.63%	80.63%
	Principal component 2	2.3205	19.34%	100.00%
Pengdun	Principal component 1	12.1934	87.10%	87.10%
	Principal component 2	1.1663	8.33%	95.43%
All three areas	Principal component 1	1.65253	41.31%	41.31%
	Principal component 2	1.30357	32.59%	73.90%
	Principal component 3	0.72385	18.10%	92.00%

Table S7. of principal component analysis in Figure 3a.

Zilingpu Samples	Principal Component Coefficient 1	Principal Component Coefficient 2	Zhanghe Samples	Principal Component Coefficient 1	Principal Component Coefficient 2	Pengdun Samples	Principal Component Coefficient 1	Principal Component Coefficient 2
Cucumber-y1	0.2725	0.22106	Eggplant-p1	0.32077	0.04093	Tomato-1	0.26751	0.32915
Cucumber-y2	0.27492	−0.19552	Eggplant-p2	0.31812	−0.09477	Tomato-2	0.27404	−0.26713
Cucumber-y3	0.25657	0.34072	Eggplant-p3	0.32146	−0.00655	Tomato-3	0.28558	−0.05572
Cucumber-y4	0.28283	0.05565	Eggplant-p4	0.2875	−0.29376	Tomato-4	0.27826	−0.20955
Tomato-g1	0.27825	−0.15371	Chili-H1	0.2673	0.36469	Cucumber-m1	0.28372	0.01392
Tomato-g2	0.27804	0.15663	Chili-H2	0.2586	0.38927	Cucumber-m2	0.1839	0.20897
Tomato-g3	0.28259	−0.06488	Chili-H3	0.21713	0.48351	Cucumber-m3	0.28367	0.11682
Chili-g1	0.28053	0.11596	Chili-H4	0.27649	0.33477	Cucumber-m4	0.11303	0.8235
Chili-g2	0.25874	0.32744	Cauliflower-1	0.29398	−0.26567	Eggplant-1	0.28384	−0.09206
Chili-g3	0.2263	0.48243	Cauliflower-2	0.28453	−0.30551	Eggplant-2	0.2842	−0.08544
Pumpkin-r1	0.27017	−0.24284	Cauliflower-3	0.31524	−0.12866	Eggplant-3	0.28229	−0.14143
Pumpkin-r2	0.25273	−0.36292	Cauliflower-4	0.28503	−0.30357	Chili-s1	0.28603	−0.03834
Pumpkin-r3	0.2536	−0.35807				Chili-s2	0.28634	−0.01293
Pumpkin-r4	0.26782	−0.26277				Chili-s3	0.2863	−0.01473

For explanation of abbreviations, see Figure 4.

Table S8. of principal component analysis in Figure 3b.

Sample	Principal Component Coefficient 1	Principal Component Coefficient 2	Principal Component Coefficient 3
Cucumber-y1-ZL	0.97964	0.35075	−0.2511
Cucumber-y2-ZL	1.41049	−0.46836	−1.03102
Cucumber-y3-ZL	0.87996	1.23695	1.03456
Cucumber-y4-ZL	1.29265	0.33583	0.55011
Tomato-g1-ZL	1.81049	−0.45808	0.01531
Tomato-g2-ZL	2.0617	0.21742	−0.3022
Tomato-g3-ZL	2.01325	−0.0787	0.64127
Chili-g1-ZL	1.41053	0.15912	−0.35222
Chili-g2-ZL	1.31557	0.45024	−0.32971
Chili-g3-ZL	1.34258	0.59929	−0.38203
Pumpkin-r1-ZL	0.51898	−0.8176	0.40052
Pumpkin-r2-ZL	0.70801	−2.72476	1.21264
Pumpkin-r3-ZL	1.0053	−3.58494	2.3408
Pumpkin-r4-ZL	0.63961	−0.73371	−0.35946
Eggplant-p1-ZH	0.27219	0.74864	0.04041
Eggplant-p2-ZH	0.24957	0.63436	−0.35888
Eggplant-p3-ZH	0.46308	0.35167	-0.65233
Eggplant-p4-ZH	0.1597	3.04697	2.00485
Chili-H1-ZH	0.88604	−0.24871	−1.20149
Chili-H2-ZH	0.66209	−0.29759	−1.34558
Chili-H3-ZH	0.78369	−0.43359	−0.81281
Chili-H4-ZH	0.78152	−0.22378	−1.21918
Cauliflower-1-ZH	−0.31152	1.91192	0.89454
Cauliflower-2-ZH	−0.30744	1.5379	0.3954
Cauliflower-3-ZH	−0.22161	1.20534	0.35996
Cauliflower-4-ZH	−0.31969	1.4991	0.35916
Tomato-1-PD	−0.85279	−0.07686	−0.73223
Tomato-2-PD	−1.02272	−1.24251	0.44231
Tomato-3-PD	−1.06577	−0.41265	−0.1411
Tomato-4-PD	−1.07822	−0.95811	1.2765
Cucumber-m1-PD	−1.45074	−0.39525	−1.09693
Cucumber-m2-PD	−1.53484	−0.41741	−1.45038
Cucumber-m3-PD	−1.39221	−0.12021	−0.57642
Cucumber-m4-PD	−1.41003	1.51807	0.49971
Eggplant-1-PD	−0.76806	−0.3036	−0.04517
Eggplant-2-PD	−0.91264	−0.29784	−0.15563
Eggplant-3-PD	−0.89122	−0.4189	−0.17728
Chili-s1-PD	−2.62989	−0.36572	0.22684
Chili-s2-PD	−2.73328	−0.38084	0.12444
Chili-s3-PD	−2.74396	−0.34386	0.15385

For explanation of abbreviations, see Figure 4.

Table 9. Pearson correlation analysis of PAEs in different samples.

	Zilingpu						Zhanghe						Pengdun					
	**DMP**	**DEP**	**BBP**	**DBP**	**DEHP**	**DOP**	**DMP**	**DEP**	**BBP**	**DBP**	**DEHP**	**DOP**	**DMP**	**DEP**	**BBP**	**DBP**	**DEHP**	**DOP**
DMP	1	0.3696	--	−0.2990	0.5339*	0.9166 **	1	−0.9875 **	--	−0.0547	0.4274	−0.8347 **	1	0.9153**	--	−0.1998	0.4670	−0.6159
DEP		1	--	−0.4606	−0.2643	0.2945		1	--	0.0960	−0.4680	0.8614 **		1	--	−0.0530	0.2222	−0.4225
BBP			--	--	--	--			--	--	--	--			--	--	--	--
DBP				1	−0.1097	−0.3078				1	0.3525	0.1738				1	−0.3156	0.6122
DEHP					1	0.6486 *					1	−0.5619					1	−0.5287
DOP						1						1						1

* Correlation is significant at 0.05 level (two-tailed); ** Correlation is significant at 0.01 level (two-tailed).

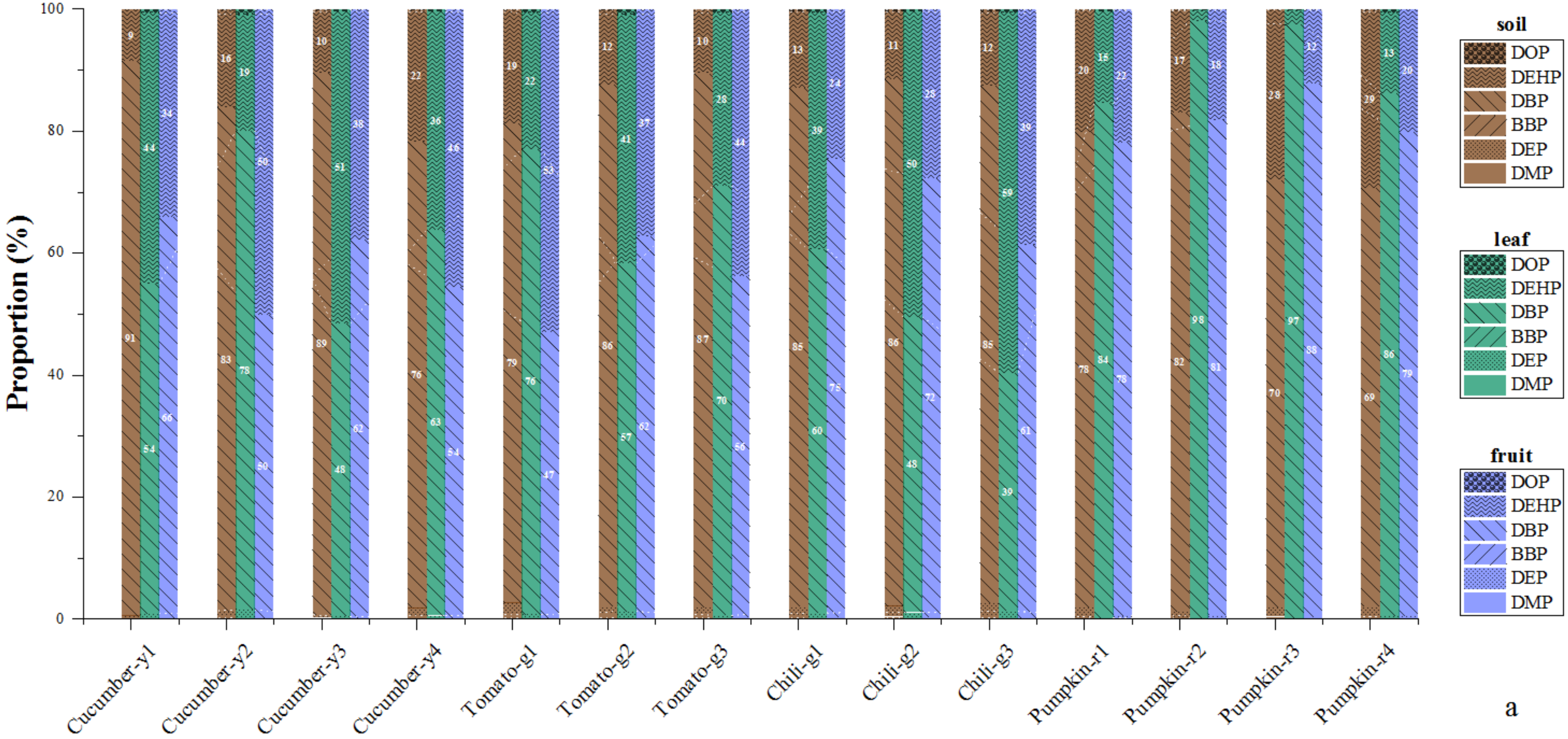
Proportion (%)
Cucumber-y1
Cucumber-y2
Cucumber-y3
Cucumber-y4
Tomato-g1
Tomato-g2
Tomato-g3
Chili-g1
Chili-g2
Chili-g3
Pumpkin-r1
Pumpkin-r2
Pumpkin-r3
Pumpkin-r4
soil
leaf
fruit
DOP
DEHP
DBP
BBP
DEP
DMP

a

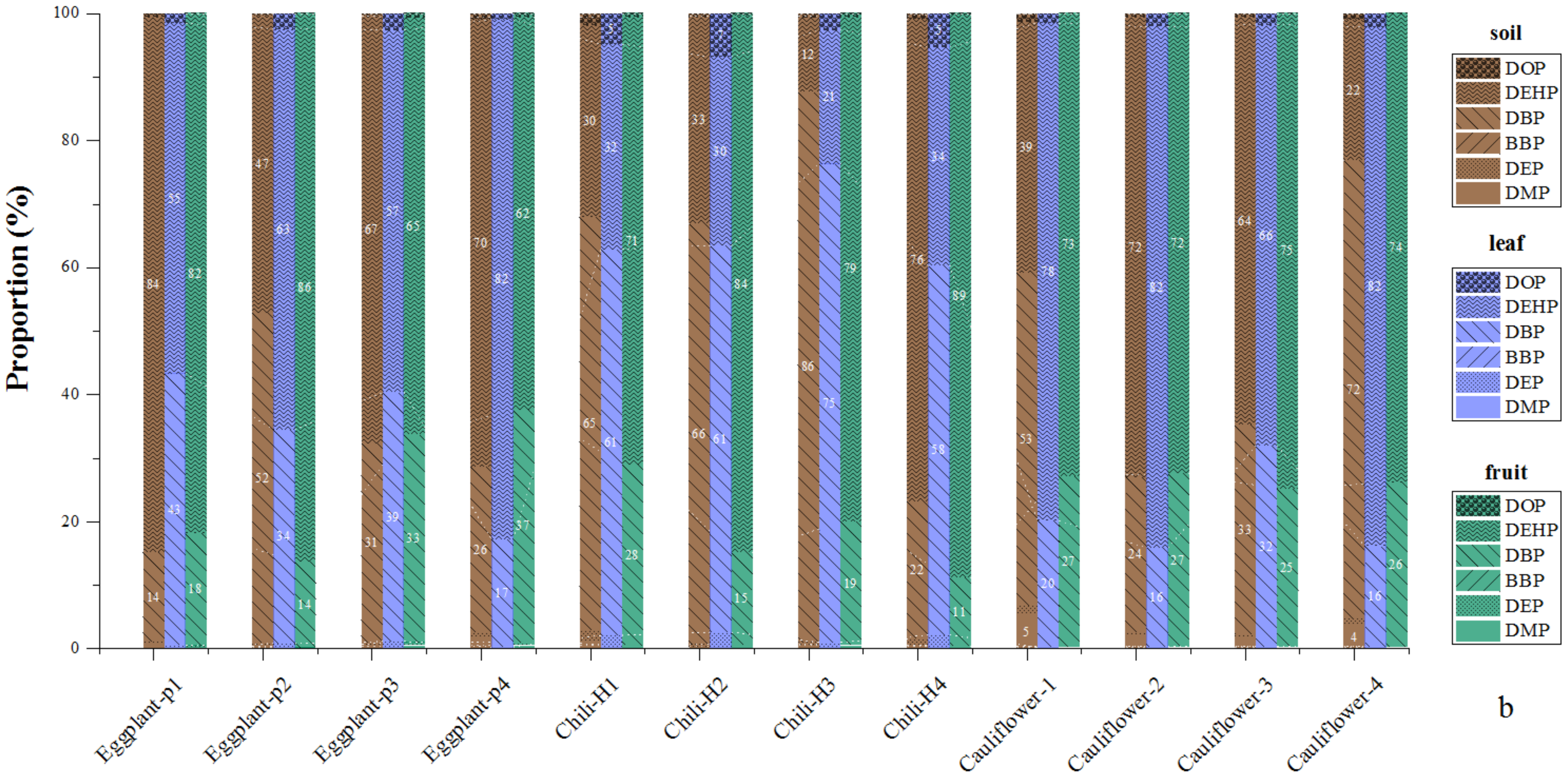
Proportion (%)
0
20
40
60
80
100
Eggplant-p1
Eggplant-p2
Eggplant-p3
Eggplant-p4
Chili-H1
Chili-H2
Chili-H3
Chili-H4
Cauliflower-1
Cauliflower-2
Cauliflower-3
Cauliflower-4
soil
DOP
DEHP
DBP
BBP
DEP
DMP
leaf
DOP
DEHP
DBP
BBP
DEP
DMP
fruit
DOP
DEHP
DBP
BBP
DEP
DMP
b

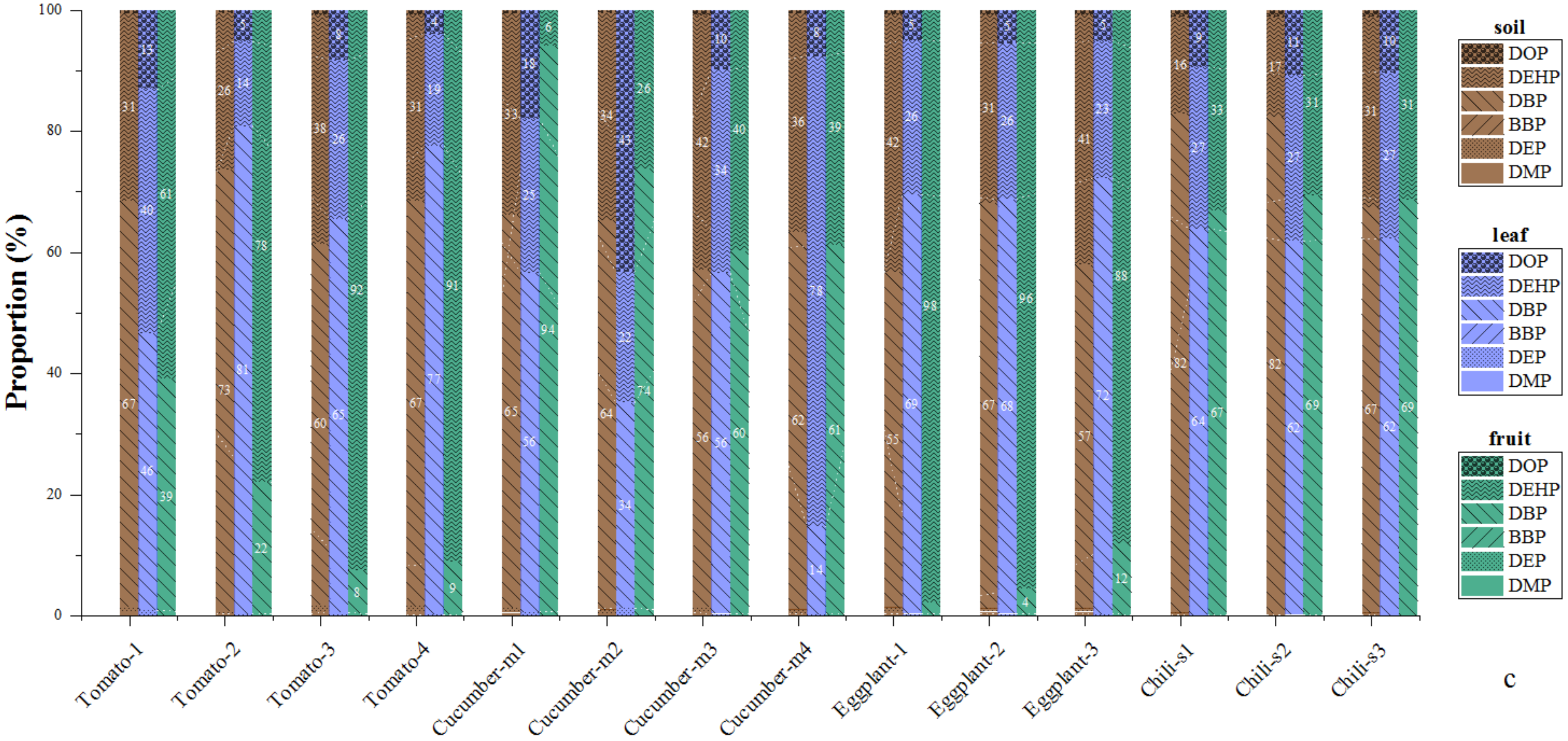

Figure 1. The composition patterns of PAEs in samples collected from (**a**) Zilingpu (*n* = 52 = 14 fruit sample + 14 leaf sample + 14 soil sample). Cucumber-y1, the first sampling greenhouse planted cucumber (yellow)/yellow cucumber; Tomato-g1, the first sampling greenhouse planted tomato (green)/green tomato; Chili-g1, the first sampling greenhouse planted chili (green)/green chili; Pumpkin-r1, the first sampling greenhouse planted pumpkin (red chestnut)/red chestnut pumpkin. (**b**) Zhanghe (*n* = 36 = 12 fruit sample + 12 leaf sample + 12 soil sample). Eggplant-p1, the first sampling greenhouse planted eggplant (purple long)/purple long eggplant; Chili-H1, the first sampling greenhouse planted chili (Hangzhou)/Hangzhou chili; Cauliflower-1, the first sampling greenhouse planted cauliflower. (**c**) Pengdun (*n* = 52 = 14 fruit sample + 14 leaf sample + 14 soil sample). Tomato-1, the first sampling

greenhouse planted tomato; Cucumber-m1, the first sampling greenhouse planted cucumber (mini)/mini-cucumber; Eggplant-1, the first sampling greenhouse planted eggplant; Chili-s1, the first sampling greenhouse planted chili (screw)/screw chili. Histogram heights <3% are hidden.
